# Reading and skimming clinical information: insights from experiments examining medical students’ eye movement behaviour

**DOI:** 10.1186/s12909-025-08412-z

**Published:** 2025-12-10

**Authors:** Marina A. Soltan, Kayleigh L. Warrington, Kiruth Sidhu, Alexander G. Roney, Victoria A. McGowan, Samuel Moffatt, Kevin B. Paterson, Colin R. Melville, Sarah J. White

**Affiliations:** 1 School of Psychology and Vision Sciences, University of Leicester, Leicester, UK; 2 Respiratory Medicine, University of Birmingham, Birmingham, UK; 3 Morecambe Bay Hospital Trust, Lancaster, UK; 4 Musgrove Park Hospital, Taunton, UK; 5 University of Manchester, Manchester, UK

**Keywords:** Reading, Skimming, Eye-movements, Cognitive processing

## Abstract

**Background:**

Reading times are shorter and comprehension is poorer during skim-reading compared to more careful reading for comprehension. Here we provide a novel examination of the effect of skimming on medical students’ reading of clinical texts. Three eye tracking experiments are reported. Each experiment manipulates the reading task (reading for comprehension, skimming for gist) and a key characteristic of the text (legibility, context, accuracy). Together the experiments provide key insights into how medical students read and skim clinical information.

**Methods:**

Participants were fourth year medical students. In each experiment participants read for comprehension and skimmed for gist. Experiment 1 examined the effect of font legibility, comparing reading behaviour for vignettes presented in a legible vs. less legible font (*n* = 28). Experiment 2 examined the effect of contextual cues, comparing reading of clinical statements that were preceded by a neutral cue vs. a cue stating the diagnosis (*n* = 28). Experiment 3 examined the integration of the text with prior knowledge by comparing reading behaviour for statements that were accurate or inaccurate (*n* = 20). Eye movements were recorded to determine how reading processes differed according to reading strategy and the text manipulations.

**Results:**

Across all three experiments skim-reading resulted in eye movement indices consistent with more superficial processing of text (shorter first-pass and re-reading times, *p*s < 0.001). There were fewer and shorter eye fixations during skimming compared to reading for comprehension (*p*s < 0.001) (Experiment 1). A less legible font was found to slow down reading (*p*s < 0.001), but did so similarly for skimming and reading for comprehension (Experiment 1). There were smaller effects of context (Experiment 2) and text accuracy (Experiment 3) for re-reading measures during skimming, indicating that skimming produces poorer integration of text with patient information or clinical knowledge.

**Conclusions:**

The eye tracking results are consistent with previous work indicating that levels of comprehension can be reduced during skim-reading. The study also demonstrates that legibility and contextual cues (e.g., diagnosis sub-headings) are important for efficient reading. Especially when learning key concepts or making key decisions, medical students and healthcare practitioners should be aware that content may be missed or only superficially processed during skimming.

**Supplementary Information:**

The online version contains supplementary material available at 10.1186/s12909-025-08412-z.

## Background

A substantial proportion of everyday reading behaviour is thought to be characterised by skim-reading [1]. Time pressures within clinical environments, and the requirement to read large amounts of text in medical education, may substantially compromise reading goals. Medical students may rush to get through required reading or skim text during exams, and clinical practitioners may quickly skim through handwritten and electronic patient notes. It has long been established that text comprehension, especially for detail within the text, is poorer during skimming compared to reading for comprehension [1–5]. Nevertheless, despite evident patient safety implications, there has been very limited objective, quantitative scientific examination of the processes involved in reading clinical information, and crucially, to our knowledge, no studies of the effects of task demands on how medical students read and comprehend.

Some studies have employed self-report measures to examine approaches to reading clinical texts. For example, medical students’ reports of their time spent reading course content [6] and physicians’ reports of how they navigate medical records [7]. Other studies have examined medical students’ reading times, for example to examine effects of report format [8]. In contrast, for non-clinical texts, many studies have employed eye movement recording methods to provide detailed insights into the mechanisms underlying reading [9]. Very few studies have employed such methodologies to examine healthcare practitioners’ reading behaviour [10–12]. For example, Krupinski et al. examined the effect of radiology report structure on eye movement behaviour [13] and Vilppu et al. examined the effect of medical expertise on eye movement behaviour during reading of patient case studies [14]. In contrast, a number of research studies have used eye movement recording methods to investigate healthcare practitioners’ examination of medical images [15] and other work has examined eye movements during viewing of videos of patient cases [16] and procedures [17].

**Fig. 1:**
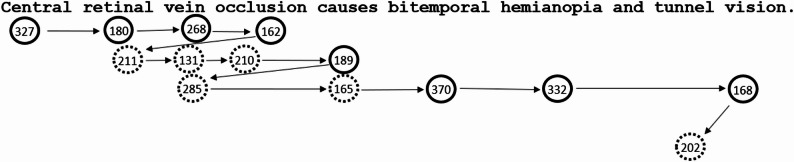
Example eye movement behaviour during reading for comprehension The circles represent eye fixation positions and the numbers indicate the fixation duration in milliseconds (ms). The arrows represent saccades. The solid line circles represent fixations that occurred during first-pass. First-pass includes all fixations from the first fixation on a word until leaving it to the left or moving to the right. First-pass time on a word is zero if that word is skipped during first-pass. The dashed circles represent fixations that occurred during re-reading. In this example, the first-pass reading time for the sentence would be 1996ms and the re-reading time for the sentence would be 1204ms. Note that re-reading time is recorded as zero when no re-reading occurs throughout the trial. Shorter re-reading times therefore reflect reduced likelihood of re-reading and/or shorter re-reading times when re-reading occurs

Eye movements can provide unique insights into what is processed when during reading. As we read, the eyes make a series of rapid movements (saccades) and pauses (fixations). Importantly, visual and oculomotor processes are closely co-ordinated with the attention and linguistic processes involved in reading, such that during reading for comprehension there is cognitive control of when and where the eyes move [18]. Figure 1 provides an example of eye movement behaviour during reading for comprehension. Note that words can be skipped (not directly fixated) and sometimes the eyes move back in the text (regress) and re-reading occurs. Previous work (with non-clinical texts) indicates that during skim-reading, words are more likely to be skipped, there are fewer and shorter fixations and there is less re-reading compared to during reading for comprehension [5, 19–21]. These behaviours may contribute to poorer comprehension during skim-reading [2–5]. For example, during skimming a larger proportion of words may not be accurately identified, and comprehension of the meaning (integration across the text and with prior knowledge) may be much more superficial. For illustration, videos of medical students’ reading for comprehension and skimming behaviour with naturalistic stimuli (clinical letters) are available to view in our Open Science Framework (OSF) repository (https://osf.io/64kcb/).

The present study takes a different approach to previous work. The experiments manipulated medical students’ task demands in order to compare different reading strategies, specifically, reading for comprehension and skimming for gist. Furthermore, in order to examine the cognitive processes involved in processing clinically related text, all three of the experiments manipulated a specific aspect of the text (legibility, context, accuracy), providing insight into how medical students process text and how different levels of text processing might be modulated by task demands. (Note that this work is distinct from studies that focus on differences in reading behaviour across individuals [22, 23].)

### Reading for comprehension vs. skimming for gist: manipulation of task demands

A fundamental aim is to examine how task demands affect medical students’ reading behaviour. In educational and clinical settings factors such as time limitations are likely to modulate reading goals and behaviour. For the experiments presented here, task demands were modulated experimentally by manipulating the participants’ instructions (read for comprehension vs. skim for gist), and by manipulating the frequency and type of questions that participants responded to (more frequent and more detailed comprehension questions during reading for comprehension). Analyses of eye movement behaviour for the clinical vignettes in Experiment 1 provide a summary of how reading strategies can affect behaviour. In line with previous work [5, 19–21], we anticipated fewer and shorter eye fixations and fewer regressive saccades during skimming. We also predicted shorter first-pass and re-reading times during skimming compared to reading for comprehension (Experiments 1, 2 and 3). (See Table 1; Fig. 1 for definitions of the outcome measures.) Shorter reading times and limited re-reading during skimming likely reflect more superficial processing of the text, in line with previous work indicating that comprehension is poorer during skim-reading [1–5]. Note that there was no direct measure of comprehension in the present study as the comprehension questions were manipulated to drive task demands (rather than for comparison of comprehension scores across conditions).

**Table 1:** Outcome measures

Analysis	Measure	Definition	Interpretation
Expt 1 vignettes (baseline legible font)	Total reading time (ms)	Time from when the text appears on the screen until the participant presses a button to move on (including, saccades, blinks etc.).	Longer total reading times may be indicative of more in-depth processing of the text or processing difficulty.
	Reading rate (wpm, words per minute)	The number of words read per minute across the text (calculation based on total reading time and number of words).	Higher reading rates may be indicative of less in-depth processing of the text.
	Number of fixations	Total number of fixations.	More fixations provide more opportunities for visually detailed samples of the text. A larger number of fixations may be indicative of more in-depth cognitive processing or processing difficulty.
	Average fixation duration (ms)	Mean duration of fixations for all eye fixations (including first-pass and re-reading fixations).	Longer fixation durations provide more time for cognitive processing of the text and may be indicative of more in-depth processing or difficulty.
	Number of regressive saccades	Eye movements that move backwards in the text (including intra-word backward refixations and inter-word regressions)	Short-range regressive saccades usually occur due to incomplete lexical processing or oculomotor error. Long-range regressions can be triggered by sentence integration difficulty. More regressive saccades are indicative of more detailed reanalysis of the text or due to text processing difficulty.
Expts 1–3 2 (task) X 2 (text manipulation, i.e., font, context or accuracy)	First-pass reading time (ms)	The sum of all of the first-pass fixation durations.	Indicative of initial text processing, including word recognition processes (orthographic, lexical, semantic) and integration of words in text context.
	Re-reading time (ms)	The sum of all of the fixation durations after first-pass (zero if there are no re-reading fixations).	Indicative of later text processing, especially sentence integration and reanalysis, including integration with prior knowledge. Limited re-reading time is indicative of limited reanalysis and integration which may be associated with more superficial comprehension.

See Figure 1 for a definition of “first-pass”, and an example of first-pass and re-reading time calculations

*Abbreviations*: *Expt* Experiment, *ms* milliseconds, *wpm* words per minute

The present study provides a novel test of how task demands modulate reading behaviour when in-depth comprehension requires integration of the text with prior (medical) knowledge. The research provides a test of whether established findings (based on reading of text that relates to general world knowledge) hold for detailed text with content that relates to readers’ in-depth prior knowledge. Understanding how task demands can affect medical students’ reading behaviour has potentially important implications for levels of comprehension in educational and professional medical settings. Skim-reading may occur in educational settings when students have limited time to work through required reading. Encouraging students to reflect on how their reading behaviour may affect their comprehension may help them to study more effectively and perform well in exams, adapting their reading strategies to optimise comprehension. Skim-reading may occur in clinical settings when there may be insufficient time to read and fully comprehend patient notes. Crucially, optimising comprehension and being more aware of what may have been missed may also improve decision making in clinical environments.

### Manipulations of text characteristics

Experimental methods were used to objectively measure, record and quantify medical students’ eye movements using an eye tracker in real time during reading of clinically related stimuli. Experiments 1, 2, and 3 each manipulates a key characteristic of the text and examines whether these effects are modulated by task demands (reading strategies).

Experiment 1 examines effects of font legibility on eye movement behaviour during reading of clinical vignettes. The vignettes were similar to those presented as a lead-in for exam questions throughout medical school, similar to shift handover notes, and similar to the notes that might be used when presenting a case to a senior decision maker. Longer reading times were predicted for the less legible font, in line with previous work [24]. Effects of text legibility are especially relevant to the use of handwritten notes [25, 26] in clinical practice.

Experiment 2 tests whether contextual (diagnosis) cues facilitate subsequent reading of clinical statements, in line with previous research demonstrating facilitative effects of context for non-clinical material [27]. Contextual cues are likely to activate specific relevant concepts within readers’ detailed prior knowledge such that these facilitate subsequent reading behaviour. The experiment has potential implications for the use of contextual cues in clinical texts, for example, diagnosis headings in referral letters.

Experiment 3 examines effects of text accuracy, comparing reading behaviour for clinical statements that are accurate or inaccurate (i.e., consistent or inconsistent with medical students’ knowledge). Longer reading times for inaccurate statements [28] is indicative of difficulty with integrating the concepts in the text with prior knowledge. Crucially, larger effects of text accuracy during reading for comprehension compared to skimming would be indicative of more superficial integration of the text with prior knowledge during skimming.

To summarise, the present study examines medical students’ eye movement behaviour during reading for comprehension and skimming for gist, as determined by the experimental manipulation of task demands (manipulation of task instructions as well as the frequency and type of comprehension questions). Effects of reading task are reported for several measures of eye movement behaviour for the clinical vignettes in Experiment 1, providing an overview of how task can modulate medical students’ reading behaviour. In total we report three experiments, each of which provides an experimental test of a specific text manipulation. Together these provide insights into the effect of font legibility (Experiment 1), contextual cues (Experiment 2) and the nature of text integration (Experiment 3) during reading for comprehension and skimming of clinically related text.

## Methods

### Participants

Participants were University of Leicester fourth year MBChB medical students’ (*n* = 76). All participants were native English speakers with normal or corrected-to-normal vision and had no history of dyslexia. Participants undertook the studies individually in the eye-tracking laboratory. Each participant completed the study in under one hour.

### Design

All three experiments had a 2 × 2 within-participant and within-item design. Stimuli materials are available in the OSF repository (https://osf.io/64kcb/). All of the experiments included a manipulation of reading task (reading for comprehension vs. skimming for gist). For all three experiments (Expts), in the reading for comprehension block a question followed every trial. The proportion of trials that were followed by a question was substantially lower in the skimming block (Expt 1: 22%; Expt 2: 20%; Expt 3: 15%). For all experiments, questions in the reading for comprehension block probed understanding of a detail within the text whereas questions in the skimming block probed understanding of the gist of the text. For Experiment 3, the questions did not require an understanding of whether the content was correct or not.

Each of the three experiments employed different text stimuli and manipulated an additional variable: *Experiment (1)* 80 vignette paragraphs of 5 lines (*n* = 28). Comparison of paragraphs presented in legible (Times New Roman) and less legible (Script MT) font. *Experiment (2***)** 64 clinical statements (e.g., “The patient presented with thirst, weight loss, tiredness and slow wound healing.”) were each preceded by either a neutral cue, where the diagnosis was blanked out with “X”s (e.g., “The patient was diagnosed with XXXXXXXXXX”), or a contextual cue stating the diagnosis (e.g., “The patient was diagnosed with diabetes.”) (*n* = 28). *Experiment (3)* 56 statements describing complex clinical concepts with a text accuracy manipulation, such that statements were either accurate (e.g., “Type 2 diabetes can cause retinopathy, neuropathy and nephropathy.”) or inaccurate (e.g., “Type 2 diabetes can cause ‘panda eyes’ due to periorbital ecchymosis.”), (consistent or inconsistent with medical students’ prior knowledge) (*n* = 20). The beginnings and ends of the sentences in Experiment 3 were spliced across items to ensure that lexical characteristics were controlled across the text accuracy conditions (see Supplementary Information). Note that the stimuli for Experiments 2 and 3 comprised single sentences in order to enable tight experimental control of the linguistic manipulations.

### Procedure

See Supplementary Information for details of the eye tracker (Eye-Link 1000) calibration procedures etc. For each experiment, participants completed two blocks of trials, one for each reading task. Half of the participants undertook the reading for comprehension block first and half undertook the skimming block first. Participants received written instructions before the experiment and before each block of trials (reading for comprehension: “read all of the sentences/text carefully”; skimming for gist: “skim the sentences quickly to understand the general content of the text”). Participants pressed a button to indicate when they had finished reading and to answer yes/no questions. In Experiment 3, participants also completed a post-experiment questionnaire that tested their prior knowledge of the clinical concepts within the items (see Supplementary Information).

### Data analysis

To broadly characterise the effect of reading task on eye movement behaviour during reading for comprehension and skimming, global measures of behaviour are reported for the Experiment 1 vignettes (baseline legible condition). For the analyses of each of the text manipulations (Expt 1: font legibility; Expt 2: contextual cues; Expt 3: text accuracy) just two summary measures are reported: first-pass reading time and re-reading time^1^.

Analyses for each measure are based on means for each participant in each condition. Datasets were tested for normality and homogeneity of variance. Statistical analyses are reported with alpha set at 0.05. The global measures in Experiment 1 were examined with paired samples *t*-tests and non-parametric tests (Wilcoxon signed rank and Sign tests). First-pass and re-reading measures in Experiments 1–3 were analysed with 2 (task: read, skim) X 2 (text manipulation) repeated measures ANOVAs. Where there were significant interactions, paired sample *t*-tests were undertaken, with the alpha level adjusted for multiple comparisons with a Bonferroni correction. Additional non-parametric tests were also undertaken (see Supplementary Information). In all cases the non-parametric test results produced the same pattern as the parametric test results. Therefore, although the parametric results should be interpreted with caution where there are violations of assumptions, the consistent pattern of results across the tests together provides clear evidence for the pattern of results described. Note that the analysis approach was adopted as it is appropriate for the simple manipulations employed here, and accessible for an inter-disciplinary audience (see Footnote iii in Supplementary Information).

See the Declarations section for information about ethical approval and data availability. Additional methodological details are provided in Supplementary Information (including full details of participant replacement, data exclusions, data analysis procedures, additional statistics such as confidence intervals, and additional measures). Analysis code and data files are available in the OSF repository (https://osf.io/64kcb/).

## Results

We first report the effects of reading task on reading time, reading rate and measures of eye movement behaviour for the clinical vignettes in Experiment 1. Together these measures provide an overview of how task demands can modulate medical students’ reading behaviour, revealing how reading behaviour differs during reading for comprehension compared with skimming for gist. We then report the effects of reading task and text manipulations on measures of first-pass reading time and re-reading time (see Table 1 for definitions) for each of the three experiments. We confirm that the effect of reading task holds for each of the measures across each of the experiments, before focusing on the effect of the specific text manipulations. We then report the effects of font legibility (Experiment 1), contextual cues (Experiment 2) and the nature of text integration (demonstrated by effects of text accuracy, Experiment 3). For each experiment, we examine whether these effects are modulated by reading task. We report if there is an interaction between task and text manipulation for each of the experiments and where significant, we explain the nature of the interaction.

### Effects of reading task (Experiment 1, baseline legible font condition)

Global measures of eye movement behaviour when reading for comprehension and skimming the vignettes (legible Times New Roman font) are reported in Table 2. Parametric (paired samples *t*-test) and non-parametric (Wilcoxon signed rank and Sign tests) results are detailed in Supplementary Information (Table S2) and all show significant effects of task for all measures (*p*s < 0.001). Total reading times were shorter and reading rates faster during skimming compared to reading for comprehension. There were fewer and shorter fixations during skimming compared to reading for comprehension. There were also fewer regressive saccades during skimming compared to reading for comprehension. The results are in line with the patterns of eye movement behaviour reported for reading for comprehension and skimming non-clinical texts [5, 19–21]. The present study demonstrates that similar patterns of behaviour also occur when medical students are reading for comprehension and skimming clinically related texts.

**Table 2:** Experiment 1, vignettes in legible font. Mean global measures of eye movement behaviour during reading for comprehension and skimming for gist

Measure	Reading for comprehension	Skimming for gist
Total reading time (ms)	21,936 (9,134)	9,171 (2,953)
Reading rate (wpm)	202 (75)	471 (207)
Number of fixations	83 (32)	38 (11)
Average fixation duration (ms)	214 (23)	193 (21)
Number of regressive saccades	27 (13)	11 (4)

Standard deviations in parentheses. Statistics calculated from participant means

*Abbreviations*: *ms* milliseconds, *wpm* words per minute

### Effects of reading task and text manipulations (Experiments 1–3)

First-pass and re-reading measures are reported in Table 3 and ANOVA results are reported in Table 4 for each of the three experiments. For all three experiments there were significant effects of task such that both first-pass and re-reading times were significantly shorter during skimming compared to reading for comprehension. These results are in line with the global results for the vignettes in legible font (Table 2). The effects of the text manipulations and interactions with reading task are summarised for each experiment below. Note that the same pattern of results is shown for non-parametric tests, reported in Supplementary Information (Table S7).

**Table 3:** Experiments 1, 2 and 3: Mean first-pass and re-reading time. Effects of task and text manipulation

Expt	Measure (ms)	Manipulation	Reading for comprehension	Skimming for gist
1	First-pass reading time	Legible	5509 (1271)	4255 (1272)
		Less legible	6252 (1359)	4917 (1380)
	Re-reading time	Legible	12,399 (7531)	3029 (1731)
		Less legible	13,483 (8127)	3881 (1974)
2	First-pass reading time	Neutral	2230 (438)	1844 (455)
		Diagnosis cue	2162 (455)	1712 (383)
	Re-reading time	Neutral	1965 (982)	467 (301)
		Diagnosis cue	1461 (828)	309 (209)
3	First-pass reading time	Accurate statement	2257 (681)	1822 (514)
		Inaccurate statement	2241 (655)	1858 (554)
	Re-reading time	Accurate statement	1702 (1003)	452 (337)
		Inaccurate statement	2112 (1099)	526 (490)

Standard deviations in parentheses. Statistics calculated from participant means

*Abbreviations*: *Expt* Experiment, *ms* milliseconds

**Table 4:** Experiments 1, 2 and 3: 2 × 2 ANOVA results for first-pass and re-reading time. Effects of task and text manipulation

Expt	Effect	First-pass reading time		Re-reading time	
		*F*	*Partial η* ^*2*^	*F*	*Partial η* ^*2*^
1	Task	49.25*	0.65	49.46*	0.65
	Font legibility	51.15*	0.65	19.46*	0.42
	Task X Font legibility	0.20	0.00	0.28	0.01
2	Task	34.64*	0.56	85.83*	0.76
	Contextual cue	15.61*	0.37	29.05*	0.52
	Task X Contextual cue	2.27	0.08	7.77*	0.22
3	Task	33.31*	0.64	47.35*	0.71
	Text accuracy	0.18	0.00	14.48*	0.43
	Task X Text accuracy	0.87	0.04	12.66*	0.40

ANOVAs were undertaken based on means for each participant for each condition (degrees of freedom: Expt 1 & 2: 1,27; Expt 3: 1,19). For datasets where there were violations of assumptions non-parametric tests were also undertaken (see Supplementary Information Table S7). The ANOVA results for effects of task and text manipulations, and the contrasts reported in the main text, produced the same pattern of statistical significance as the non-parametric tests

*Abbreviations*: *Expt* Experiment, *F* ANOVA *F* statistic, *Partial η*^*2*^ Partial Eta Squared

* *p* < 0.05

*Experiment 1*: There were significant effects of font for both first-pass and re-reading time, such that reading times were longer for less legible compared to legible font. There were no interactions, indicating that the effect of font was similar for the two reading tasks. The results indicate that during both reading for comprehension and skimming, initial reading and later re-reading behaviour are both affected by text legibility. Note that a range of factors could contribute to the longer reading times, including factors such as visual complexity and familiarity with the script. Crucially, the results indicate that medical students require more time to read text that is presented in a less legible format, in line with previous work that employed non-clinical texts [24].

*Experiment 2*: There were significant effects of contextual cue for both first-pass and re-reading time. Reading times were longer when the cue was neutral compared to when the diagnosis was specified before the statement about the symptoms and signs. This pattern is consistent with previous work demonstrating facilitative effects of context for non-clinical text [27]. For first-pass reading time there was no interaction between task and contextual cue. Re-reading times were a lot shorter during skimming compared to reading for comprehension, and there was a significant interaction between task and contextual cue. Contrasts revealed reliable effects of context for re-reading times for both reading for comprehension and skimming (*p*s < 0.025) (see Supplementarty, Tables S6 and S7). The interaction indicates that the effect of the contextual cue was larger for reading for comprehension. The results indicate that the provision of the diagnosis cue facilitated subsequent first-pass and re-reading of the statement about the symptoms and signs during both reading for comprehension and skimming. For example, the cue may have facilitated recognition of related words in the sentence or facilitated subsequent integration of the sentence with existing knowledge of the disorder. The interaction indicates that the cue especially facilitated subsequent processing of the statement about symptoms and signs during reading for comprehension. The larger effect for reading for comprehension may arise because the text was processed to a greater depth, such that the diagnosis cue especially facilitated integration of the sentence with readers’ prior knowledge of the disorder.

*Experiment 3*: There was no reliable effect of text accuracy for first-pass reading time and no interaction with task. There was a significant effect of text accuracy for re-reading time and an interaction. Contrasts indicate that for reading for comprehension there was a reliable effect of text accuracy on re-reading times such that re-reading times were longer when the statements were inaccurate compared to accurate (*p* < 0.025). In contrast, there was no significant effect of text accuracy for re-reading times during skimming (*p* = 0.185) (see Additional File 1, Tables S6 and S7). The results indicate that during reading for comprehension, the medical students attempted to integrate the meaning of the sentences with their existing knowledge. Consequently, when the sentences were inaccurate, they experienced difficulty integrating the text with their understanding of the clinical issue, such that their re-reading times were longer when the sentence was inaccurate compared to when it was accurate (in line with previous research with non-clinical texts [28]). In contrast, when skimming the results indicate that integration of the text with prior knowledge is much more superficial, such that re-reading times were much shorter and not significantly affected by the accuracy of the text. Note that both Experiments 2 and 3 revealed interactions with task demands for the re-reading measure. Together the results indicate that reduced re-reading during skimming may be especially reflective of more superficial text processing, for example, with more limited integration across the text and with prior knowledge.

## Discussion

The study provides novel experimental insights into how medical students process clinical texts. The analyses of reading measures for the clinical vignettes in Experiment 1 demonstrate that medical students’ skimming behaviour is characterised by shorter reading times, fewer and shorter eye fixations and fewer regressive saccades compared to reading for comprehension. The analyses of effects of the text manipulations demonstrate that reading times are longer for less legible fonts (Expt 1), that reading (especially more careful reading) is facilitated by contextual cues (diagnosis cue, Expt 2) and that re-reading times are longer during reading for comprehension when text content conflicts with detailed prior knowledge (Expt 3). The smaller effects of context on re-reading time during skimming (Expt 2) and the absence of reliable effects of text accuracy (Expt 3) during skimming indicate that text processing can be more superficial during skimming of clinically related text. That is, integration across clinical texts and with prior clinical knowledge may be more limited during skimming compared with reading for comprehension.

The study builds on previous work that has employed eye movement methodologies to examine reading behaviour for generic [9] and clinically related [10–14] texts. The patterns of behaviour involved in reading for comprehension and skimming are similar to those reported for non-clinical texts [5, 19–21]. The findings are in line with previous work indicating that levels of comprehension are reduced during skim-reading [1–5]. Overall, the experiments provide novel insights into the effects of task demands on medical students’ reading strategy and cognitive processes. Potential practical implications of the findings are set out below, along with suggestions for further research. For example, we set out suggestions for testing approaches that may maximise comprehension in medical education and clinical environments. Finally, we note the limitations of the studies presented here and summarise key points that may be relevant for undertaking further work in this area.

### Implications and future directions

The results have potentially very important implications for scenarios that require comprehension of clinically related text. In Experiment 3, medical students did not take extra time to read incorrect clinical statements when skimming, indicating that they were not properly integrating the content with their medical knowledge. Skim-reading may limit text integration, especially if there is limited re-reading. In educational contexts skim-reading may limit the depth of learning. When setting assigned reading, it may be valuable for medical teachers to reflect on how much content medical students will be able to read carefully within their available study time, such that they can achieve a good level of accurate comprehension [6]. For those taking exams, key details may be missed if exam materials are not read carefully before responding to questions. In clinical contexts skim-reading may impair subsequent decision making. Superficial processing of medical information in a clinical context could result in key information being missed, or minimal critical analysis and integration, potentially leading to errors, such as incorrect diagnosis or inappropriate treatment (key to patient safety and clinical care).

Future studies may examine in more detail the effect of skimming under time pressure on comprehension and decision making. Note that different reading strategies may also be reflective of the standards of coherence [29] that readers adopt for comprehension, which may vary depending on readers’ goals and the demands of the task. Further work could examine whether medical students and professionals may benefit from better awareness [30, 31] of the limitations of rapid reading and the standards of coherence for comprehension that they employ. Reflecting on how reading behaviour may affect comprehension may enable students to identify gaps in their learning and make more informed decisions, such as querying potential key details that may have been missed. Improving medical students’ and healthcare practitioners’ ability and inclination to reflect realistically on their level of text comprehension may enable more accurate insight into their level of understanding. It may be valuable to highlight these issues within study skills (and professional skills) guidance for medical students. More broadly, there may be implications for patient safety and quality of care, ensuring that sufficient time is made available for reading relevant notes etc. prior to critical decision making. Future studies may also employ experimental methods to examine the optimal format for clinical documentation that facilitates accurate comprehension of key details even when there is limited time to read.

The effects of font legibility and context have important implications for clinical documentation and digital medicine. In line with previous work with generic texts [24], Experiment 1 shows that medical students take longer to read less legible clinical texts, which may restrict the amount of material that can be read when time is limited. Comprehension may therefore be more efficient for clearly legible text, for example, compared with less legible handwritten clinical notes. Note that up to 10% of UK NHS trusts have yet to adopt electronic patient records [32]. Any omissions (or errors) in readers’ comprehension of clinical notes may impact on communication with colleagues (for example, at shift handover, or in the presentation of cases to senior decision makers). Also, in line with the wider literature [27], the results of Experiment 2 indicate that the provision of contextual (diagnosis) cues can facilitate subsequent reading. One possibility is that the cues may activate relevant specific concepts in the healthcare practitioners’ (and healthcare trainees’) prior clinical knowledge, facilitating lexical identification of specialised words and integration with prior clinical knowledge. For example, including the diagnoses at the start of a referral letter may facilitate subsequent reading of the case details. Other key information presented at the start of letters and notes, such as a medication list, may also provide valuable context for comprehension of more detailed text. Future studies could examine patients’ reading of medical or health information leaflets and websites. Issues such as font legibility (e.g., font and font size), time pressures and superficial reading goals may also have key implications for patients’ comprehension and appropriate management of their conditions.

### Limitations and generalisability

A wide range of task demands and text characteristics involved in naturalistic reading likely generate a broad spectrum of different reading behaviours. For example, medical students and healthcare practitioners may sometimes scan with very high reading rates (skipping many consecutive words or lines of text) in order to identify relevant sections that may then be read more carefully. In naturalistic contexts there are likely to be a broad range of individual, text-related and environmental factors that contribute to skimming. For example, short reading times may occur due to time pressure [5] or when readers are not engaging in in-depth or active comprehension [33]. In the present study participants were reading tightly controlled stimuli in laboratory conditions. Future studies may examine in more detail a broader range of task demands and reading strategies that are likely to occur in clinical environments. The eye-tracking methods employed here provide valuable insights into the naturalistic time course of comprehension processes. However, it is always valuable to have converging evidence from different paradigms and future studies could also employ other methods, such as accuracy scores, to provide direct measures of comprehension. Further studies may also examine different populations and manipulations, as outlined below.

The participants in the present study were fourth year medical students. The patterns reported here are likely to be reflective of the reading behaviour of similar samples of medical students early in their professional careers. Similar patterns may also be observed for more senior healthcare practitioners, however some aspects may differ, for example, due to their expertise and greater familiarity with the clinical content. Ideally further studies would be undertaken with more senior healthcare practitioners, however recruiting sufficiently large samples of these individuals is likely to be challenging given their work schedules and location (especially for in-person eye tracking studies). Further work may also be required to examine the reading behaviour of medical students and healthcare practitioners who differ from the sample tested here, for example, those with a history of reading difficulty (dyslexia) or those who are reading in a second language [34].

The present study employs only a small number of eye movement measures to provide an overview of the effect of text manipulations on reading behaviour (first-pass and re-reading times). Future studies may benefit from employing a broader range of measures that may help reveal more subtleties, and build a more detailed account of the time course of effects [9]. For example, in Experiment 3, there were smaller effects of text accuracy during skim-reading compared to during reading for comprehension. Subsequent studies may manipulate the accuracy of a critical word, and detailed reading measures for that critical word may reveal a more complex pattern of results. Furthermore, future studies with greater power (more stimuli items and participants) may be sensitive to more subtle effects. For example, there may be small but significant effects of accuracy during skim-reading (e.g., effects of accuracy on the likelihood of making regressions out of a critical word for both reading and skimming, but still a greater level of rereading during reading for comprehension compared to skimming). Nevertheless, note that the effect sizes for some of the manipulations reported here are quite large, however future studies with more subtle manipulations (or greater variability in behaviours) may require much larger sample sizes [35]. Recruiting and testing large samples of medical students for eye tracking experiments can be challenging given their busy timetables and placements.

## Conclusion

The study employed eye movement and cognitive science methods to examine how task demands can modulate medical students’ processing of clinical texts. Compared to reading for comprehension, during skimming medical students employed fewer and shorter fixations, and fewer regressive eye movements. Reading times were longer for less legible texts and reading was facilitated by contextual cues. The results show that re-reading times are much shorter during skimming and the effects of text accuracy indicate that integration of the text when skimming can be limited. Future work may examine in more detail how skim-reading in educational contexts and clinical situations may limit comprehension and affect outcomes. For example, how medical students’ reading strategies affect their learning, how task demands such as time limitations may impair subsequent clinical decision making and potential adverse impacts on patient safety and quality of care. Overall, further research in this area may have considerable benefits for medical education, patient safety, clinical care, digital medicine and clinical documentation.

## Supplementary Information

Supplementary Material 1

## Data Availability

The materials and datasets generated during the current study are available in the Open Science Framework (OSF) repository, https://osf.io/64kcb/.

## Abbreviations

Expt: Experiment
ms: milliseconds
wpm: words per minute
OSF: Open Science Framework

## Footnotes

1 In Experiment 1 the stimuli were vignettes that included multiple sentences whereas in Experiments 2 and 3 the stimuli comprised a single sentence clinical statement. Consequently, first-pass and re-reading times are much longer in Experiment 1 compared to Experiments 2 and 3. For the single sentences in Experiments 2 and 3, re-reading time could be very short, and re-reading did not always occur. In contrast in Experiment 1 the re-reading times are calculated across the vignette and re-reading tends to account for a larger proportion of reading time compared to the single sentences. For example, participants could skip from the first line to the fifth line, then back to the second line, such that all reading behaviour on lines two, three and four of the vignette would be classified as re-reading.
