# Supplementary material for: Reading and skimming clinical information: insights from experiments examining medical students’ eye movement behaviour
Source: BMC Med Educ. 2025 Dec 10;26:76. doi: 10.1186/s12909-025-08412-z (PMC12801452; doi:10.1186/s12909-025-08412-z)
Supplement: Supplementary file 1 — Supplementary Material 1 [file 12909_2025_8412_MOESM1_ESM.pdf]

Additional File 1: Supplementary materials

Reading and skimming clinical information:

Insights from experiments examining medical students' eye movement behaviour

Marina A. Soltan, Kayleigh L. Warrington, Kiruth Sidhu, Alexander G. Roney, Victoria A. McGowan,

Samuel Moffatt, Kevin B. Paterson, Colin R. Melville, Sarah J. White

Methodology and data analysis: Supplementary details

Participants

The mean age of participants was ~23 years (Expt 1: *M* = 23.2; *SD* = 1.74; Expt 2: *M* = 23.0, *SD* = 3.4; Expt 3: *M* = 23.7yrs, *SD* = 2.7). Prior to each experiment, participants were screened for visual acuity at the viewing distance (80cm) using an ETDRS chart[1]. For all three experiments, participants received a £10 honorarium[i] to compensate for their time. Participants for all three experiments were self-selected. None participated in more than one experiment. Participants responded to study adverts (emails, posters, lecture shout-outs). Intended sample sizes were achieved.

Stimuli

Lists of experimental stimuli for each experiment are available in the Open Science Framework (OSF) repository. Additional details are provided below for each experiment. For all three experiments items were counterbalanced across conditions using a Latin square design. For each experiment, an equal number of participants were randomly assigned to each Latin square list. The order of the items within each block was randomised. Each block of experimental items was

[i] Participants received the honorarium regardless of their performance (the honorarium was not an incentive). Nevertheless, it is not possible to rule out that differences across studies may arise when participants are compensated in different ways.

preceded by a set of practice items (number per block: Expt 1: 5; Expt 2: 15; Expt 3: 8). For each of the experiments participants also read or skimmed additional text that was presented either before each block, or inter-mixed with the experimental trials in each block[ii]. Text was presented in black on a light grey background.

Experiment 1: Each paragraph was composed of five lines of text with a maximum of 80 characters per line. The legible font, Times New Roman, was presented in 19pt bold font. The less legible font, Script MT, was presented in 20pt bold font. The font sizes were selected to ensure that the characters were on average approximately the same size. 3.8 characters subtended one degree of visual angle on average. Lines of text were double spaced and areas of interest (corresponding to each word) extended above and below each line. The distance between the edge of the line of text to the interest area boundary above or below subtended 0.93 degrees of visual angle. Together the line spacing, the size of the regions of interest, and the spatial eye tracking accuracy (<0.55 degrees of visual angle), minimised issues related to accuracy across lines due to vertical drift[2] (no manual or algorithm-based adjustments were made for vertical drift).

Experiment 2: Sentences were presented in Courier New bold font. 3.3 characters subtended one degree of visual angle.

[ii] For Experiment 1, blocks of trials were preceded by the same participants reading for comprehension or skimming 12 fictional naturalistic clinical letters. Example videos of participants' eye movement behaviour when reading for comprehension or skimming these letters are provided in the Open Science Framework (OSF) repository. In line with normal practice, Experiments 2 and 3 included additional filler items that were intermixed with the experimental stimuli within each block of trials. For Experiment 2 there were 32 filler trials in each block, these were similar to the experimental trials except that the contextual cue was either neutral, or provided a category for the type of statement that followed (Cause, Symptoms, Investigation or Management). For Experiment 3 there were 26 filler trials in each block, these were straightforward medically related statements, adapted from information on the NHS choices "Health A-Z" website. Note that the eye movements of an additional sample of novice (first year) medical students also completed Experiment 3. As expected, for this novice sample there were no effects of text accuracy. For brevity and for consistency across the three studies presented here, only data for the fourth-year medical students is reported for Experiment 3.

Experiment 3: Pairs of items were generated such that sentence accuracy was manipulated by either retaining the original sentences (accurate) or by splicing the two parts of the sentences together (inaccurate), as shown in the example items below.

Accurate condition:

Base of skull fractures cause 'panda eyes' due to periorbital ecchymosis.

Type 2 diabetes can cause retinopathy, neuropathy and nephropathy.

Inaccurate condition:

Base of skull fractures cause retinopathy, neuropathy and nephropathy.

Type 2 diabetes can cause 'panda eyes' due to periorbital ecchymosis.

The questions after the sentences required readers to recall what the sentences referred to (e.g., "*Did the sentence refer to a type of fracture?*"), but did not require an understanding of whether the sentence was correct or not.

An additional eight fourth year medical students (who did not participate in the experiment but who were from the same population) rated the sentences for their grammatical flow (disregarding their semantic content) on a scale of 1-5 ("1" = the sentence does not flow, "3" = the flow is a bit awkward, "5" = the flow appears natural). There was no significant difference between the sentence ratings for the accurate (M = 4.78, SD = 0.282) and inaccurate (M = 4.74, SD = 0.294) conditions (ts < 1.01, ps > 0.3).

After completing the eye tracking experiments, each participant undertook a post-experiment questionnaire which they filled in electronically on a spreadsheet. The questionnaire tested their prior knowledge of the clinical concepts within the items. For each sentence, participants indicated if they were confident that the sentence was accurate, confident that the sentence was inaccurate, or "don't know / unsure". Prior knowledge scores provided a test of whether the participants had prior knowledge of most of the concepts (see data exclusion details below).

Sentences were presented in Courier New font in black on a very light grey background. 3.3 characters subtended one degree of visual angle.

Apparatus

For all three experiments, eye movements were recorded with an EyeLink 1000 SR Research Ltd. eye tracker. The eye tracking laboratory was located within the School of Psychology and Vision Sciences at the University of Leicester, UK. Viewing was binocular though only movements of the right eye were recorded. Pupil location was sampled at a rate of 1000Hz with spatial accuracy <0.55 degrees of visual angle for the vignette stimuli (Experiment 1) and <0.3 degrees of visual angle for the single line sentence stimuli (Experiments 2 and 3). (Spatial accuracy limits ensured quality of measurement and reliability of data collectors). The eye tracker was calibrated at the start of the experiment (Expt 1: 9-point calibration; Expts 2 and 3: 3-point calibration), checked before every trial, and recalibrated when necessary. Movement was minimised using a chinrest and forehead rest.

Procedure

Each experiment comprised two blocks of trials. Each participant completed the study (including instructions, consent, both experimental blocks, breaks and debrief) in under one hour. Note that participants took longer to complete the block of trials that involved reading for comprehension compared to the block that involved skimming for gist. For Experiment 3, the debrief made clear to participants that some of the materials were inaccurate.

Data exclusions

For each experiment a small number of participants and trials had to be removed. For all of the experiments, trials were excluded if total reading times were <500ms, as such short response times are likely caused by participants accidentally pressing the button to finish the trial (Expt 1: 1 trial excluded; Expt 2: 2 trials excluded: Expt 3: 0 trials excluded). Each experiment also had additional data exclusions:

Experiment 1: Two participants were replaced due to eye tracking data collection difficulties. 13 trials were omitted from one participant's dataset (participant 16, reading for comprehension block) due to a technical issue that resulted in the eye tracking data not being saved for the last 13 trials in the block. Some trials included a few eye fixations on areas of the screen other than the paragraph (outside of the interest areas). Trials were excluded if more than five eye fixations occurred outside of the interest areas. No participants had more than ten trials excluded for this reason. Overall four percent of trials were excluded.

Experiment 2: One participant was replaced due to eye tracking data collection difficulties.

Experiment 3: After Experiment 3, participants completed a questionnaire (the prior knowledge test, see Stimuli section above) that tested their knowledge of the clinical concepts within the experimental items. One item was removed due to low accuracy scores across all participants. Participants were replaced if more than fifteen percent of the inaccurate sentences were categorised as correct (four participants were replaced).

For all experiments, any fixations shorter than 80ms or longer than 1,200ms were discarded. Outliers were removed by trimming data points >2.5SDs above the mean for each participant and each condition for each measure (see the R code in the OSF repository).

Data analysis

Analyses were undertaken using R[3]. All analyses are based on means for each participant in each condition[iii]. The Shapiro Wilk test (R function "shapiro.test") was used to assess if the data was

[iii] The present study adopted an analysis approach that is appropriate given the simple manipulations, and also likely to be accessible to an inter-disciplinary audience. Future studies that employ more subtle manipulations with larger sample sizes could employ alternative techniques that include participants and stimuli items as random variables (mixed effects models[5]).

normally distributed in each condition. For the analyses of effects of task for the global measures in Experiment 1 (comparison of two conditions) the *F* test (R function "var.test") was used to assess homogeneity of variance. For the first-pass and re-reading time measures (four conditions) in Experiments 1-3, homogeneity of variance was assessed using the Bartlett test (R function "bartlett.test").

For the Experiment 1 global measures, Table S1 reports descriptives including measures such as confidence intervals to demonstrate precision of the estimates. Note that the global measure of total reading time is calculated from when the text appears on the screen until the participant presses a button to move on. Total reading time therefore includes first-pass and re-reading time (Tables S3 and S4), but also time for eye movements (saccades), eye fixations away from the text (outside of the interest areas) and time for eye blinks. Note also that some participants had shorter or longer reading times, such that the distribution of reading times, rates and number of fixations / regressions were not normally distributed, and variance differed across the two conditions (see Table S2). For completeness, both parametric (paired samples *t*-test) and non-parametric (Wilcoxon signed rank and Sign tests) are reported for all of the global measures (see Table S2). The paired samples *t*-tests (two-tailed) were undertaken using the R function "t.test" and Cohen's d effect sizes were calculated using the "cohens_d" function in the "effectsize" package. The analyses are based on means for each participant in each condition (t_1 analyses). Additional analyses were undertaken based on means for each stimulus item per condition[4] (t_2 analyses) (these analyses produced the same pattern of results, details omitted for brevity). The Wilcoxon signed rank test was undertaken using the R function "wilcox.task". The effect size was calculated using the rank-biserial correlation (the R function "rank_biserial" in the "effectsize" package). Note that for some conditions the distribution of difference scores was not symmetrical, hence Sign tests were also undertaken. The Sign tests were undertaken on the difference scores between the two condition means, using the R function "binom.test". Where there are violations of the assumptions, the results should be interpreted with caution. Nevertheless, the consistent pattern of results across the tests, and the

large effect sizes, together provide clear evidence for an effect of task on the global measures of eye movement behaviour.

Table S1. Experiment 1 vignettes in legible font: Global measures of eye movement behaviour during reading for comprehension and skimming. Mean (Standard Error and 95% confidence interval in parentheses) and median (interquartile range in parentheses). Statistics calculated from participant means.

Measure	Mean (*SE*) (95% confidence interval)		Median (IQR)	
	Read	Skim	Read	Skim
Total reading time (ms)	21,936 (1,726)<br>(18,394 - 25,478)	9,171 (558)<br>(8,026 - 10,316)	20,327<br>(15,057 - 26,172)	9,594<br>(7,418 - 11,282)
Reading rate (wpm)	202 (14)<br>(173 - 231)	471 (39)<br>(391 - 552)	195<br>(145 - 253)	382<br>(345 - 506)
Number of fixations	83 (6)<br>(71 - 96)	38 (2)<br>(33 - 42)	72<br>(61 - 102)	39<br>(31 - 46)
Average fixation duration (ms)	214 (4)<br>(205 - 223)	193 (4)<br>(185 - 201)	210<br>(199 - 230)	193<br>(181 - 209)
Number of regressive saccades	27 (2)<br>(22 - 32)	11 (1)<br>(9 - 12)	22<br>(17 - 36)	10<br>(8 - 13)

Notes:

The interquartile range (IQR) is reported as the 25th (Q1) and 75th (Q3) percentiles of the data.

Table S2. Experiment 1 vignettes in legible font: Effects of task shown for the paired samples *t*-test, Wilcoxon signed rank test and the sign test. All analyses based on means across participants.

Measure	Paired samples *t*-test (*df* = 27)				Wilcoxon signed rank		Sign test
	t	*p*	95% CI	Effect size (Cohen's d)	*p*	Effect size (r_{rb})	*p*
Total reading time (ms) [1,3,4]	8.51	< 0.001	9687 - 15842	1.61	< 0.001	0.94	< 0.001
Reading rate (wpm) [2,3,4]	-7.48	< 0.001	-343 - -196	-1.41	< 0.001	-0.93	< 0.001
Number of fixations [1,3,4]	8.71	< 0.001	35 - 56	1.65	< 0.001	0.95	< 0.001
Average fixation duration (ms)	8.62	< 0.001	16 - 25	1.63	< 0.001	0.46	< 0.001
Number of regressive saccades [1,3,4]	7.86	< 0.001	12 - 20	1.48	< 0.001	0.88	< 0.001

Notes:

r_{rb} = rank biserial correlation.

[1] The Shapiro Wilk test (tests if the data is normally distributed) for the Read condition was significant.
[2] The Shapiro Wilk test (tests if the data is normally distributed) for the Skim condition was significant.
[3] The *F* test (test for homogeneity of variance) was significant.
[4] The distribution of difference scores was not symmetrical.

Descriptives for effects of task and text manipulation in the three experiments are reported in Tables S3 and S4, including confidence intervals to demonstrate precision of the estimates. 2X2 repeated measures ANOVA results are reported in manuscript Table 4 and Table S5. The ANOVAs were computed using the R function “aov” and Partial Eta Squared was calculated using the “eta_squared” function in the “effectsize” package. These analyses are based on means for each participant in each condition (F_1 analyses). Additional analyses were undertaken based on means for each stimulus item per condition (F_2 analyses) to ensure that the same pattern held. The pattern of results for the F_2 analyses were the same as for the F_1 analyses (omitted here for brevity). Where there were interactions, post-hoc contrasts were undertaken using paired samples t-tests (two-tailed) with the R function “t.test” (Table S6). The superscript numbers in Tables S3 and S4 specify where violations of assumptions occurred. For these measures the non-parametric test results are presented in Table S7. Non-parametric test results are reported for effects of task and the manipulation (Expt 1: legibility; Expt 2: context; Expt 3: accuracy) (collapsing across the other variable). Table S7 also includes non-parametric test results for the post-hoc contrasts. The results for the non-parametric tests are in-line with the parametric test results. Therefore, although the ANOVA results should be interpreted with caution where there are violations of the assumptions, the consistent pattern of results across the tests together provides clear evidence for the pattern of results described in the manuscript.

Table S3. First-pass reading time measures for Experiments 1-3. Mean (Standard Error and 95% confidence interval in parentheses) and median (interquartile range in parentheses). Statistics calculated from participant means.

Expt	Manipulation	Mean (SE) (95% confidence interval)		Median (IQR)	
		Read	Skim	Read	Skim
1	Legible	5509 (240)<br>(5016 - 6002)	4255 (240)<br>(3762 - 4748)	5375<br>(4575 - 6341)	4341<br>(3549 - 4766)
	Less legible	6252 (257)<br>(5724 - 6779)	4917 (261)<br>(4382 - 5452)	6031<br>(5255 - 7443)	5219<br>(4407 - 5648)
2	Neutral[1]	2230 (83)<br>(2060 - 2400)	1844 (86)<br>(1668 - 2021)	2212<br>(1971 - 2495)	1747<br>(1561 - 2108)
	Diagnosis[1]	2162 (86)<br>(1986 - 2339)	1712 (72)<br>(1563 - 1860)	2163<br>(1843 - 2529)	1616<br>(1500 - 1871)
3	Accurate[1]	2257 (152)<br>(1938 - 2575)	1822 (115)<br>(1582 - 2063)	2222<br>(1784 - 2591)	1641<br>(1532 - 1915)
	Inaccurate	2241 (146)<br>(1935 - 2548)	1858 (124)<br>(1599 - 2118)	2186<br>(1748 - 2502)	1669<br>(1507 - 2095)

Notes:
The interquartile range is reported as the 25th (Q1) and 75th (Q3) percentiles of the data.
[1] The Shapiro Wilk test (tests if the data is normally distributed) was significant in the Skim condition.

Table S4. Re-reading time measures for Experiments 1-3. Mean (Standard Error and 95% confidence interval in parentheses) and median (interquartile range in parentheses). Statistics calculated from participant means.

Expt	Manipulation	Mean (SE, 95% confidence interval)		Median (IQR)	
		Read	Skim	Read	Skim
1	Legible[1]	12,399 (1423)<br>(9478 - 15319)	3029 (327)<br>(2358 - 3700)	10,750<br>(6356 - 16442)	2583<br>(1690 - 4144)
	Less legible[1]	13,483 (1536)<br>(10332 - 16635)	3881 (373)<br>(3115 - 4646)	10,113<br>(7628 - 17824)	3596<br>(2245 - 5009)
2	Neutral[1]	1965 (186)<br>(1584 - 2345)	467 (57)<br>(351 - 584)	1851<br>(1299 - 2390)	434<br>(294 - 600)
	Diagnosis[1,2]	1461 (156)<br>(1140 - 1782)	309 (40)<br>(228 - 390)	1267<br>(718 - 1915)	271<br>(157 - 400)
3	Accurate[1,2]	1702 (224)<br>(1232 - 2171)	452 (75)<br>(294 - 610)	1308<br>(948 - 2152)	316<br>(225 - 574)
	Inaccurate[2]	2112 (246)<br>(1597 - 2626)	526 (110)<br>(297 - 756)	1790<br>(1280 - 3054)	377<br>(178 - 657)

Notes:
The interquartile range is reported as the 25th (Q1) and 75th (Q3) percentiles of the data.
For the re-reading time measures in Experiments 1, 2 and 3 the Bartlett test (test for homogeneity of variance) was significant.
[1] The Shapiro Wilk test (tests if the data is normally distributed) for the Read condition was significant.
[2] The Shapiro Wilk test (tests if the data is normally distributed) for the Skim condition was significant.

Table S5. Experiments 1, 2 and 3: 2X2 ANOVA results for first-pass and re-reading time. Effects of task and text manipulation. Duplicate of Table 4 in main text, with *p* values.

Expt	Effect	First-pass reading time			Re-reading time		
		F	*p*	*Partial* η^2	*F*	*p*	*Partial* η^2
1	Task	49.25	<.001	0.65	49.46	<.001	0.65
	Legibility	51.15	<.001	0.65	19.46	<.001	0.42
	Task X Legibility	0.20	0.662	0.00	0.28	0.603	0.01
2	Task	34.64	<.001	0.56	85.83	<.001	0.76
	Context	15.61	<.001	0.37	29.05	<.001	0.52
	Task X Context	2.27	0.144	0.08	7.77	<.01	0.22
3	Task	33.31	<.001	0.64	47.35	<.001	0.71
	Text accuracy	0.18	0.68	0.00	14.48	<.01	0.43
	Task X Text accuracy	0.87	0.364	0.04	12.66	<.01	0.40

Expt = Experiment. *F* = ANOVA *F* statistic. *Partial* η^2 = Partial Eta Squared.

Note: ANOVAs were undertaken based on means for each participant for each condition (degrees of freedom: Expt 1 & 2: 1,27; Expt 3: 1,19).

Table S6. Experiments 2 and 3: Post-hoc Paired samples *t*-test results for re-reading time in Experiments 2 and 3.

Expt	Contrast	*t*	*p*	95% CI	Effect size (Cohen's d)
2	Read: Effect of context	4.29	<0.001	263 - 744	0.81
	Skim: Effect of context	4.16	<0.001	80 - 236	0.79
3	Read: Effect of accuracy	-4.17	<0.001	-616 - -204	-0.93
	Skim: Effect of accuracy	-1.37	0.185	-187 - 39	-0.31

Note: *t*-tests were undertaken based on means for each participant for each condition (degrees of freedom: Expt 2: 27; Expt 3: 19).
Bonferroni correction: Critical *p* value: 0.025

Table S7. Non-parametric test results for first-pass and re-reading time for datasets that violated the ANOVA assumptions (see Tables S3 and S4).

Expt	Measure	Effect	Wilcoxon signed rank test		Sign test
			p	Effect size (r_{rb})	*p*
1	Re-reading time	Task[1]	< 0.001	0.92	< 0.001
		Legibility[1]	< 0.001	-0.12	< 0.001
2	First-pass reading time	Task	< 0.001	0.56	< 0.001
		Context	< 0.001	0.14	< 0.01
	Re-reading time	Task[1]	< 0.001	0.92	< 0.001
		Context[1]	< 0.001	0.21	< 0.001
		Read: Effect of context[1]	< 0.001	0.35	< 0.01
		Skim: Effect of context[1]	< 0.001	0.34	< 0.001
3	First-pass reading time	Task[1]	< 0.001	0.38	< 0.001
		Accuracy[1]	0.609	0.03	0.824
	Re-reading time	Task[1]	< 0.001	0.86	< 0.001
		Accuracy[1]	< 0.001	0.10	< 0.01
		Read: Effect of accuracy[1]	< 0.001	0.25	< 0.01
		Skim: Effect of accuracy[1]	0.261	0.00	0.824

Notes:

r_{rb} = rank biserial correlation.

[1] The distribution of difference scores was not symmetrical.

Non-parametric results are not reported for the first-pass reading time measure in Experiment 1 as there were no violations of assumptions. For all other measures, non-parametric tests are reported for effects of task and the manipulation (legibility, context, accuracy) (collapsing across the other variable). Additional non-parametric tests were undertaken for the re-reading time contrasts in Experiment 2 and Experiment 3, to confirm the interactive pattern of effects.

Additional measures

Note that additional measures were also analysed but have been omitted from the manuscript for brevity (for example, reported in Kiruth Sidhu's BSc Medical Research (Intercalated) and Alexander Roney's MSc (Intercalated) Medical Research dissertations). The datafiles for Experiments 2 and 3 (available on the OSF project page) include two additional measures that may be of interest: The proportion of words skipped during first-pass and the total number of inter-word regressions. The means for each of these measures are reported in Table S8. ANOVA results indicate that for both experiments the proportion of words skipped was greater during skimming compared to during reading for comprehension and the number of regressions was greater during reading compared to skimming (*p*s < 0.05), in line with previous work. In Experiment 2 there were fewer regressions when the diagnosis cue was provided ($p < 0.05$, especially during reading for comprehension) and in Experiment 3 there were more regressions when the sentences were inaccurate ($p < 0.05$).

Table S8. Additional measures for Experiments 2 & 3. Mean (Standard Error) proportion of first-pass word skips and total number of inter-word regressions. Measures calculated per participant per sentence, statistics calculated from participant means.

Expt	Manipulation	Mean proportion first-pass word skips (SE)		Mean total number of inter-word regressions (SE)	
		Read	Skim	Read	Skim
2	Neutral	0.26 (0.01)	0.35 (0.01)	3.98 (0.44)	1.53 (0.23)
	Diagnosis	0.28 (0.01)	0.35 (0.01)	3.25 (0.39)	1.34 (0.23)
3	Accurate	0.30 (0.02)	0.34 (0.02)	3.30 (0.35)	1.31 (0.17)
	Inaccurate	0.31 (0.02)	0.34 (0.02)	3.87 (0.36)	1.55 (0.22)

Comprehension questions

For all three experiments, comprehension question accuracy results were high for both reading for comprehension (Expt 1: $M = 87.0$; $SD = 6.8$; Expt 2: $M = 90.9$; $SD = 3.6$; Expt 3: $M = 94.1$; $SD = 5.0$) and skimming (Expt 1: $M = 92.4$; $SD = 7.7$; Expt 2: $M = 90.1$; $SD = 10.9$; Expt 3: $M = 98.8$; *SD*

= 5.4). Note that for all three experiments there were fewer questions in the skimming block and the questions were easier, hence the comprehension rates should be interpreted cautiously (and statistical tests for effects of reading task would not be appropriate).

Supplementary materials: References

1. Ferris FL, Bailey I. Standardizing the measurement of visual acuity for clinical research studies: Guidelines from the Eye Care Technology Forum. Ophthalmol. 1996;103;181–182.

2. Carr JW, Pescuma VN, Furlan M, et al. Algorithms for the automated correction of vertical drift in eye-tracking data. Behav Res Methods. 2022;54;287–310.

3. R Core Team (2024). R: A Language and Environment for Statistical Computing. R Foundation for Statistical Computing, Vienna, Austria.

4. Clark HH. The language-as-fixed-effect fallacy: A critique of language statistics in psychological research. J Verbal Learn Verbal Behav. 1973;12;335-359.

5. Baayen R. H., Davidson D. J., Bates D. M. Mixed-effects modeling with crossed random effects for subjects and items. J Mem Lang. 2008;59:390–412.
